# A Cellular Pathway Involved in Clara Cell to Alveolar Type II Cell Differentiation after Severe Lung Injury

**DOI:** 10.1371/journal.pone.0071028

**Published:** 2013-08-05

**Authors:** Dahai Zheng, Gino V. Limmon, Lu Yin, Nicola H. N. Leung, Hanry Yu, Vincent T. K. Chow, Jianzhu Chen

**Affiliations:** 1 Interdisciplinary Research Group in Infectious Diseases, Singapore-Massachusetts Institute of Technology Alliance for Research and Technology, Singapore; 2 Institute of Bioengineering and Nanotechnology, Agency for Science, Technology and Research, Singapore; Department of Physiology & Mechanobiology, National University of Singapore, Singapore; 3 Human Genome Laboratory, Department of Microbiology, Yong Loo Lin School of Medicine, National University of Health System, National University of Singapore, Singapore; 4 The Koch Institute for Integrative Cancer Research and Department of Biology, Massachusetts Institute of Technology, Cambridge, Massachusetts, United States of America; Centre of Influenza Research, The University of Hong Kong, Hong Kong

## Abstract

Regeneration of alveolar epithelia following severe pulmonary damage is critical for lung function. We and others have previously shown that Scgb1a1-expressing cells, most likely Clara cells, can give rise to newly generated alveolar type 2 cells (AT2s) in response to severe lung damage induced by either influenza virus infection or bleomycin treatment. In this study, we have investigated cellular pathway underlying the Clara cell to AT2 differentiation. We show that the initial intermediates are bronchiolar epithelial cells that exhibit Clara cell morphology and express Clara cell marker, Scgb1a1, as well as the AT2 cell marker, pro-surfactant protein C (pro-SPC). These cells, referred to as pro-SPC^+^ bronchiolar epithelial cells (or SBECs), gradually lose Scgb1a1 expression and give rise to pro-SPC^+^ cells in the ring structures in the damaged parenchyma, which appear to differentiate into AT2s via a process sharing some features with that observed during alveolar epithelial development in the embryonic lung. These findings suggest that SBECs are intermediates of Clara cell to AT2 differentiation during the repair of alveolar epithelia following severe pulmonary injury.

## Introduction

The lung is a branching structure of trachea, bronchioles and alveoli. In the mouse, Clara cells are the major cell type of bronchiolar epithelia and express secretoglobin family 1A member 1 (Scgb1a1 or Clara cell secretory protein). Alveolar type I (AT1s) and type II (AT2s) cells are the major cell types in the alveolar epithelia. Whereas AT1s express podoplanin (PDPN), AT2s express pro-surfactant protein C (pro-SPC) [1]–[3].

Clara cells are known to be capable of self-renewal for the maintenance and repair of bronchioles [1]–[3]. Based on resistance to naphthalene treatment, a subset of Clara cells was identified as variant Clara (Clara^v^) cells. Clara^v^ cells reside at either the bronchioalveolar duct junctions (BADJs) or neuroendocrine bodies (NEBs) and function as progenitor cells for the repair of bronchiolar epithelia as shown by pulse-chase DNA labeling [4], [5]. Some BADJ-associated naphthalene-resistant Scgb1a1-expressing cells also express pro-SPC. These cells, termed as bronchioalveolar stem cells (BASCs) [6], were shown to differentiate into both Clara cells and AT2s *in vitro* [6]. By lineage tracing in mice with a gene targeted Scgb1a1-CreER system, recent studies provided definitive evidence that Clara cells in bronchioles can self-renew and give rise to ciliated cells for the long-term maintenance and repair of lung airway [7].

Chemical-induced lung injury has been widely used to study the repair of alveoli. Earlier studies have shown that AT2s can self-renew and give rise to AT1s [8]–[11]. However, a recent study using a pro-SPC promoter-driven CreER based lineage tracing system to follow AT2s in mice showed that after bleomycin treatment, majority of the newly generated AT2s are not derived from pre-existing AT2s, indicating that other progenitor cells are involved in alveolar regeneration [12]. By tracing Scgb1a1-expressing cells in the lung of the Scgb1a1-targeted CreER mice, Rock et al showed that Scgb1a1-expressing cells can give rise to AT1s and AT2s during the regeneration of alveoli following bleomycin treatment [13]. Consistently, using the same Scgb1a1-targeted CreER lineage tracing system, we found that Scgb1a1-expressing cells also give rise to AT1s and AT2s during the repair of alveolar damage induced by influenza virus infection [14]. In the Scgb1a1-CreER lineage tracing system, the majority of labelled cells are Clara cells, although BASCs and some AT2s can also be labelled [7]. Through quantitative analyses, we provide strong evidence showing that the majority of regenerated AT2s are most likely derived from Clara cells following severe pulmonary injury induced by either bleomycin or influenza virus infection [14].

In the present study we investigate differentiation pathway through which Clara cells give rise to AT2s following severe pulmonary injury. By immunoflourescent staining, we observe a new cell type that resides in the bronchioles and expresses both Scgb1a1 and pro-SPC. We refer these pro-SPC^+^ bronchiolar epithelial cells as SBECs. Further kinetic analyses and lineage tracing show that SBECs are intermediates during Clara cell to AT2 differentiation. We also document that this differentiation process shares some features of alveolar epithelial development in the embryonic lung. Identification of a differentiation pathway by which Clara cells give rise to AT2s provides further support for regeneration of AT2s from Clara cells during the repair of alveolar damage. These findings may open new possibilities for medical interventions to promote the repair of pulmonary damage.

## Results

### SBECs are Induced in Response to Severe Alveolar Damage

**Figure 1:**
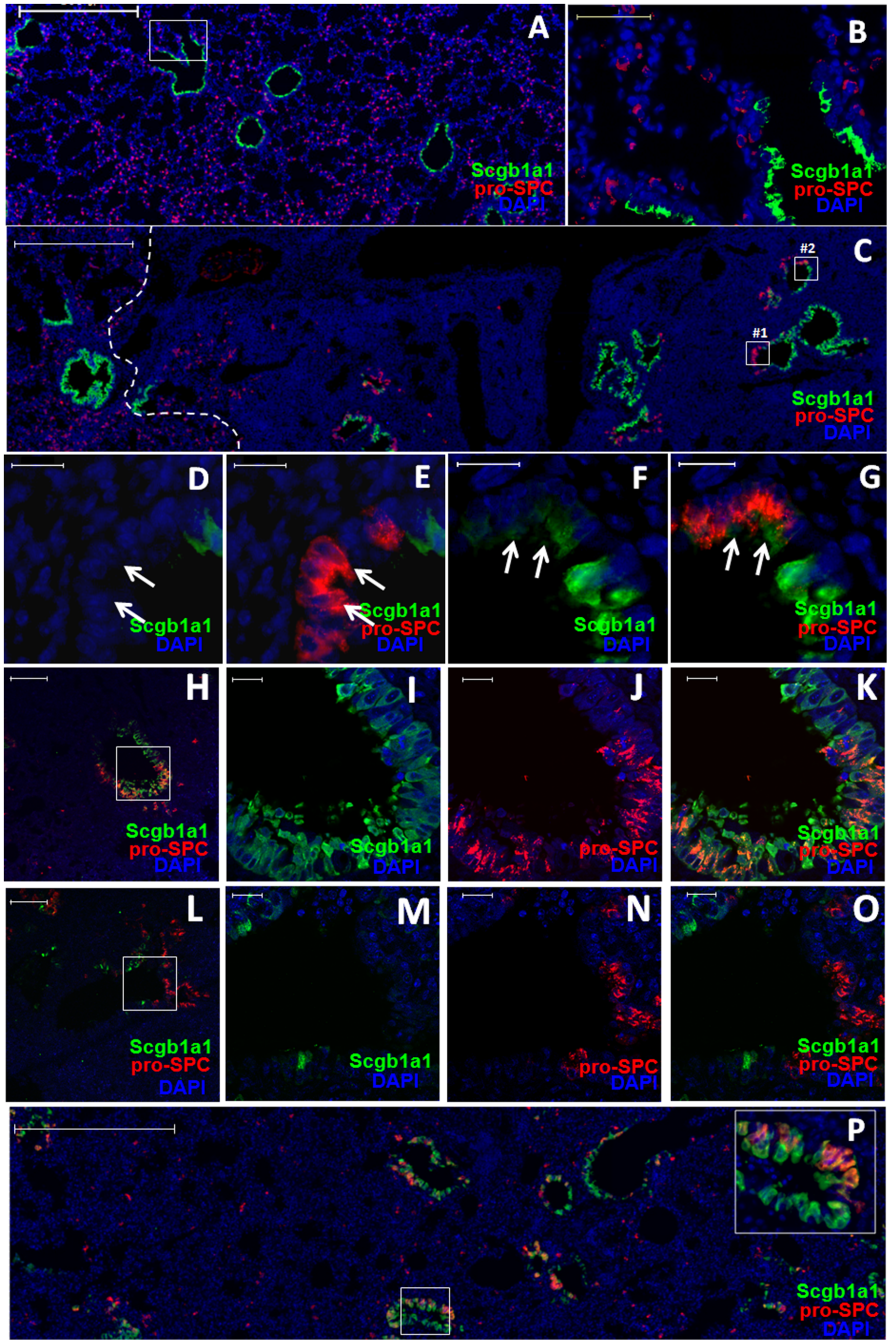
SBECs are induced following severe alveolar damage. A–C. Representative Scgb1a1 (green), pro-SPC (red) and DAPI (blue) staining of lung sections of (A) uninfected mice or (C) influenza virus-infected mice at 14 dpi. Higher magnification of the selected area in (A) is shown as (B). The white broken line in (C) demarcates the infiltrated area (to the right) and the normal area (to the left). D&E. Higher magnification of the selected area #1 in (C) either (D) without or (E) with the pro-SPC channel. Arrows indicate pro-SPC single-positive cells. F&G. Higher magnification of the selected area #2 in (C) either (F) without or (G) with the pro-SPC channel. Arrows indicate Scgb1a1 and pro-SPC double-positive cells. H-O. Representative confocal images of Scgb1a1 (green), pro-SPC (red) and DAPI (blue) staining of lung tissue sections of mice at 14 dpi. Higher magnifications of the boxed areas in H and L are shown as I–K and M-O, respectively. Scgb1a1^+^ (H–K) and Scgb1a1^−^ (L–O) SBECs are shown. P. Representative Scgb1a1 (green), pro-SPC (red) and DAPI (blue) staining of lung sections of mice at day 7 post-bleomycin treatment. High magnification of the boxed area is depicted at the upper-right corner. Scale bars: (A,C,P) 500 µm; (H,L) 100 µm; (B) 50 µm; (D–G, I–K, M–O) 20 µm.

To study how lung tissue damage is repaired, we developed a model of influenza virus-induced pulmonary injury and repair (see Figure S1 and Supplementary Materials, File S1, for details). In the normal mouse lung, staining of the Clara cell marker Scgb1a1 was confined to bronchiolar epithelia while staining of the AT2 cell marker pro-SPC was restricted to alveoli (Figure 1A,B). Following influenza virus infection, Clara cells and AT2s were severely depleted from the infected lung areas where infiltration by inflammatory cells was evident by dense DAPI staining of nuclei (Figure 1C and Figure S1). Surprisingly, we frequently observed cells that expressed the AT2 marker pro-SPC, but displayed the columnar shape of Clara cells in the bronchiolar epithelia at 14 days post infection (dpi) (Figure 1C–G). We refer these cells as pro-SPC^+^ bronchiolar epithelial cells (or SBECs). Some of the SBECs were positive for Scgb1a1, while others were negative for Scgb1a1 (Figure 1D–G). The presence of both Scgb1a1^+^ and Scgb1a1^−^ SBECs was further confirmed by confocal microscopic analysis (Figure 1H–O). Similarly, SBECs were also frequently observed in the damaged areas of the lung 7 days following bleomycin treatment (Figure 1P), which induces depletion of AT2s and AT1s from alveolar epithelia without significantly affecting Clara cells [15].

Unlike influenza virus infection and bleomycin treatment, naphthalene treatment is known to deplete Clara cells from the bronchiolar epithelia without significantly depleting alveolar epithelial cells (Figure S2A) [16]. By 9 and 12 days post-treatment, the remaining Clara cells in bronchioles were actively proliferating to repair the bronchiolar epithelial damage (Figure S2C,D). However, throughout the 14 days following treatment, no SBECs were observed. Only occasionally were Scgb1a1 and pro-SPC double positive cells detected at the BADJs of terminal bronchioles (Figure S2B and S2E). These cells are likely BASCs given that they were weakly positive for pro-SPC [6]. Taken together, these results show that SBECs are induced following severe alveolar damage caused by influenza infection or bleomycin treatment.

### SBECs Differentiate from Scgb1a1^+^ to Scgb1a1^−^ during the Course of Lung Damage Repair

**Figure 2:**
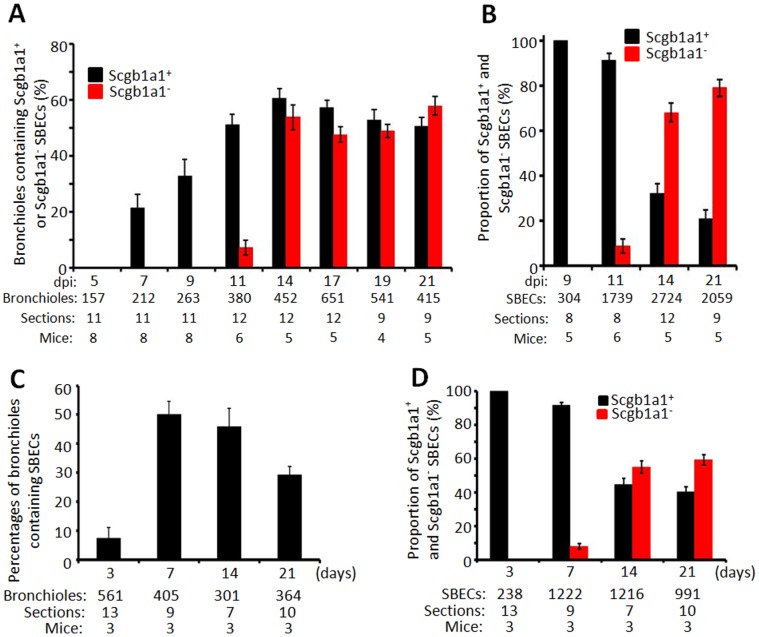
SBECs differentiate from Scgb1a1^+^ to Scgb1a1^−^ phenotype during the course of lung damage repair. A. Percentages (means ± S.E.) of bronchioles in the infiltrated area containing Scgb1a1^+^ (black bars) and Scgb1a1^−^ (red bars) SBECs. B. Proportions of Scgb1a1^+^ and Scgb1a1^−^ SBECs in a given lung section at the indicated dpi. Results are expressed as means ± S.E. C. Percentages (means ± S.E.) of bronchioles containing SBECs at the indicated days post-bleomycin treatment. D. Proportions of Scgb1a1^+^ and Scgb1a1^−^ SBECs in a given lung section at the indicated days post-bleomycin treatment. Results are expressed as means ± S.E. The numbers in (A–D) indicate the number of SBECs, bronchioles, lung sections, and mice from which the data were obtained.

To determine the relationship between Scgb1a1^+^ and Scgb1a1^−^ SBECs, we quantified these cells over time following influenza virus infection. No SBECs were detected at 5 dpi (Figure 2A), but they became detectable 7 dpi. However, all of them were Scgb1a1^+^ at 7 and 9 dpi. By 11 dpi, Scgb1a1^−^ SBECs started to become detectable, and their proportion increased with time (Figure 2A,B). By 14 and 21 dpi, most SBECs were Scgb1a1^−^ (Figure 2B). Notably, the decrease in the proportion of Scgb1a1^+^ SBECs over time was correlated with the increase in the proportion of Scgb1a1^−^ SBECs. Similarly, following bleomycin treatment, SBECs were detected as early as 3 days post treatment in approximately 8% of bronchioles, and they were all Scgb1a1^+^ (Figure 2C,D). By 7 days post-treatment, SBECs were detected in 50% of bronchioles, and most of them were still Scgb1a1^+^. By 14 and 21 days, more than half of SBECs had lost Scgb1a1 expression (Figure 2C,D). The sequential induction of SBECs that were initially Scgb1a1^+^ and then Scgb1a1^−^ suggests that SBECs differentiate from Scgb1a1^+^ to Scgb1a1^−^ phenotype during the course of lung damage repair.

### SBECs are Derived from Clara Cells

**Figure 3:**
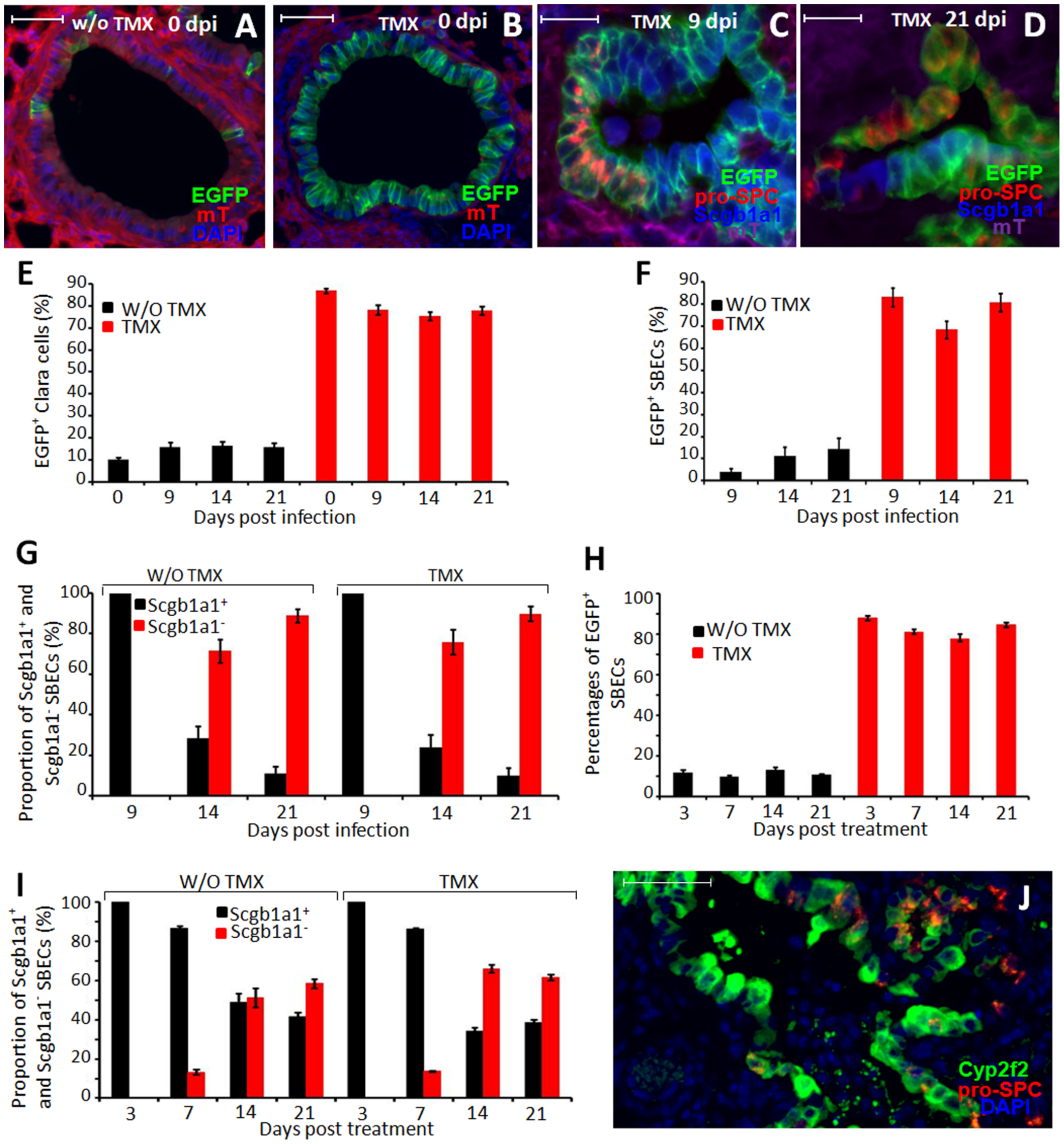
SBECs are derived from Clara cells. A&B. Representative images of bronchioles of Scgb1a1-CreER:ACTB-mT-EGFP transgenic mice without (A) or with (B) tamoxifen treatment (no infection). Expression of EGFP (green) and tomato red (mT, red) are shown. Sections were counterstained with DAPI (blue). C&D. Scgb1a1-CreER:ACTB-mT-EGFP transgenic mice were given tamoxifen, and then infected with influenza virus. Shown are representative images of lung sections analyzed for expression of EGFP (green) and tomato red (purple), and stained for pro-SPC (red) and Scgb1a1 (blue) at (C) 9 or (D) 21 dpi. E. Percentages (means ± S.E.) of Clara cells per bronchiole that express EGFP in Scgb1a1-CreER:ACTB-mT-EGFP transgenic mice without (black columns) or with (red columns) TMX treatment at various time-points post-infection. Data at each time-point were obtained by counting 5541 to 11012 Clara cells (Scgb1a1^+^ but pro-SPC^-^) from at least 15 lung sections of 4–5 mice. F. Percentages (means ± S.E.) of SBECs that express EGFP in Scgb1a1-CreER:ACTB-mT-EGFP transgenic mice without (black columns) or with (red columns) TMX treatment at different dpi. G. Proportion of SBECs that are Scgb1a1^+^ (black columns) or Scgb1a1^−^ (red columns) in Scgb1a1-CreER:ACTB-mT-EGFP transgenic mice without or with TMX treatment at different dpi. Data for (F) and (G) were obtained by counting 490 to 1774 cells from at least 16 sections of 4–5 mice per time point. H. Percentages (means ± S.E.) of SBECs that express EGFP in Scgb1a1-CreER:ACTB-mT-EGFP transgenic mice without (black columns) or with (red columns) TMX treatment at different days after bleomycin treatment. I. Proportions (means ± S.E.) of SBECs that are Scgb1a1^+^ (black columns) or Scgb1a1^−^ (red columns) in Scgb1a1-CreER:ACTB-mT-EGFP transgenic mice without or with TMX treatment at different days post-bleomycin treatment. Data for (H) and (I) were obtained from counting 351 to 1323 SBECs (pro-SPC^+^) from at least 6 lung sections of at least 2 mice per time point. J. Representative image of Cyp2f2 (green), pro-SPC (red) and DAPI (blue) staining of lung sections of mice at 14 days post-bleomycin treatment. Scale bars: (A–D) 100 µm; (j) 50 µm.

Based on their columnar shape, initial expression of Scgb1a1 and localization in the bronchioles, SBECs are likely derived from Clara cells. To directly test this, we performed lineage tracing using Scgb1a1-CreER:ACTB-mT-EGFP double transgenic mice [7]. In this transgenic system, the CreER is expressed in Scgb1a1^+^ cells but retained in the cytoplasm. Only upon binding to tamoxifen (TMX) is the CreER translocated to the nucleus where it catalyzes recombination to delete the tomato red (mT) transgene. Thus, in the absence of TMX treatment, all transgenic cells, including Scgb1a1^+^ Clara cells, express mT [17]. Upon TMX treatment, Clara cells lose mT expression and become EGFP^+^. Consistent with the previous report [7], without TMX treatment, no EGFP^+^ cells were found in the alveolar region, and only a small fraction (∼10%) of Clara cells in the bronchioles were EGFP^+^ (Figure 3A,E). With TMX treatment, approximately 85% of Clara cells within the bronchioles were EGFP^+^ (Figure 3B,E). Thus, the transgenic system has a favorable dynamic range of induction (∼8-fold) of EGFP^+^ Clara cells upon TMX treatment.

To determine if SBECs are derived from Clara cells, Scgb1a1-CreER:ACTB-mT-EGFP transgenic mice were given TMX to label Clara cells, and then infected with influenza virus to induce SBECs. Nine, 14 and 21 dpi, lung sections were stained for pro-SPC and Scgb1a1 and visualized for EGFP and mT. In the control mice that were infected but not given TMX, the fraction of EGFP^+^ Clara cells remained relatively low (∼15%) at 9, 14 and 21 dpi (Figure 3E). The slight increase (from 10% to 15%) likely reflects the expansion of pre-existing EGFP^+^ Clara cells following bronchiolar epithelial damage [7]. Supporting this interpretation, EGFP^+^ Clara cells in the bronchioles tended to cluster together (Figure S3). With TMX treatment, the fraction of EGFP^+^ Clara cells (∼80%) was as high as in TMX-treated but non-infected mice (Figure 3E). Thus, most Clara cells in the transgenic mice were labeled with EGFP following TMX treatment and infection. Correspondingly, more than 75% of SBECs were EGFP^+^ in TMX-treated and infected mice, whereas less than 12% of SBECs were EGFP^+^ in untreated but infected mice (Figure 3F). As in the wild-type mice, all SBECs in the transgenic mice were Scgb1a1^+^9 dpi, and the fraction of Scgb1a1^−^ SBECs increased to more than 70% by 14 and 21 dpi (Figure 3C,D,G).

Similarly, following bleomycin treatment, if transgenic mice were not given TMX, only ∼9% of the SBECs were EGFP^+^ (Figure 3H). If transgenic mice were given TMX, ∼80% of the SBECs were EGFP^+^. All SBECs were Scgb1a1^+^ in transgenic mice at 3 days post-bleomycin treatment, whereas 50–60% of the SBECs became Scgb1a1^−^ in TMX-treated transgenic mice by day 14 and 21 post treatment (Figure 3I). These results clearly show that SBECs are derived from Clara cells, and progress from Scgb1a1^+^ to Scgb1a1^−^ phenotype.

Clara^v^ cells are negative for Cyp2f2 (cytochrome P450, family 2, subfamily f, polypeptide 2) and are thought to regenerate other Clara cells [4], [5], [18]. Co-staining of pro-SPC and Cyp2f2 revealed that most of the SBECs were Cyp2f2^+^14–21 days post infection (Figure S4A,B) or bleomycin treatment (Figure 3J), suggesting that SBECs are not Clara^v^ cells.

### SBECs Likely give Rise to Pro-SPC^+^ Cells in the Ring Structures

**Figure 4:**
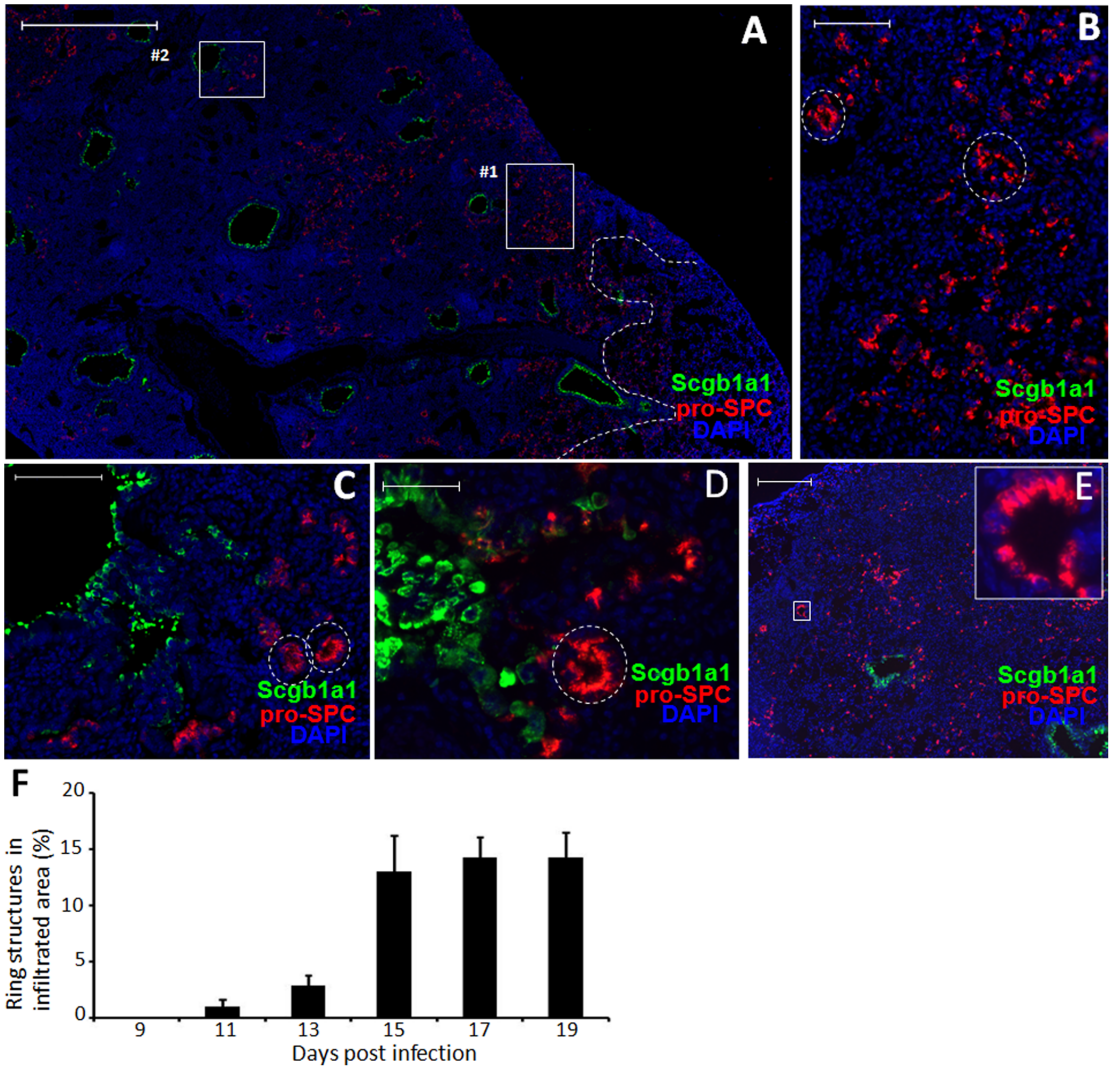
Scgb1a1^−^ SBECs likely give rise to the pro-SPC^+^ ring structures and clusters in the damaged parenchyma. A–C. Representative images of pro-SPC (red), Scgb1a1 (green) and DAPI (blue) staining of lung sections of B6 mice at 17 dpi. Higher magnification of the selected areas #1 and #2 in (A) are shown as (B) and (C), respectively. The white broken line in (A) demarcates the infiltrated area (to the left) and the normal area (to the right). D&E. Representative images of Scgb1a1 (green), pro-SPC (red) and DAPI (blue) staining of lung sections of B6 mice at 21 days post-bleomycin treatment. High magnification of the boxed area in (E) is depicted at the upper-right corner. Circles in (B–D) point to pro-SPC^+^ ring structures. F. Frequency of ring structures in the infiltrated areas of the lung at different dpi. Data (means ± S.E.) at each time-point were obtained from 7 to 15 lung sections of 3 to 8 mice. Scale bars: (A) 1000 µm; (B,C) 100 µm; (E) 200 µm; (D) 50 µm.

Next, we investigated differentiation of Scgb1a1^−^ SBECs during the regeneration of alveolar epithelia. We noticed many pro-SPC^+^ cells in the ring structures in the damaged parenchyma (Figure 4A–C). One possibility is that the ring structures are generated as a result of rapid proliferation of pro-SPC^+^ cells. To test this possibility, we pulsed infected mice 7–19 dpi with BrdU for two hours to label actively proliferating cells. As shown in Figure S5A, many Scgb1a1^+^ cells in bronchioles were labeled by BrdU, consistent with Ki67 staining (Figure S1I). In contrast, only rarely were pro-SPC^+^ cells in the damaged parenchyma or ring structures labeled by BrdU (Figure S5B–D), indicating that the generation of the ring structures is not due to proliferation of pro-SPC^+^ cells. Another possibility is that the ring structures are a result of sectioning Scgb1a1^−^ SBECs in the bronchioles at different positions (Figure 4C). Consistent with this possibility, few ring structures were observed in the damaged parenchyma at 11 dpi, but they became more abundant after 15 dpi (Figure 4F), coinciding with the development of Scgb1a1^−^ SBECs (Figure 2B). Pro-SPC^+^ ring structures were also observed following bleomycin treatment, (Figure 4D,E).

**Figure 5:**
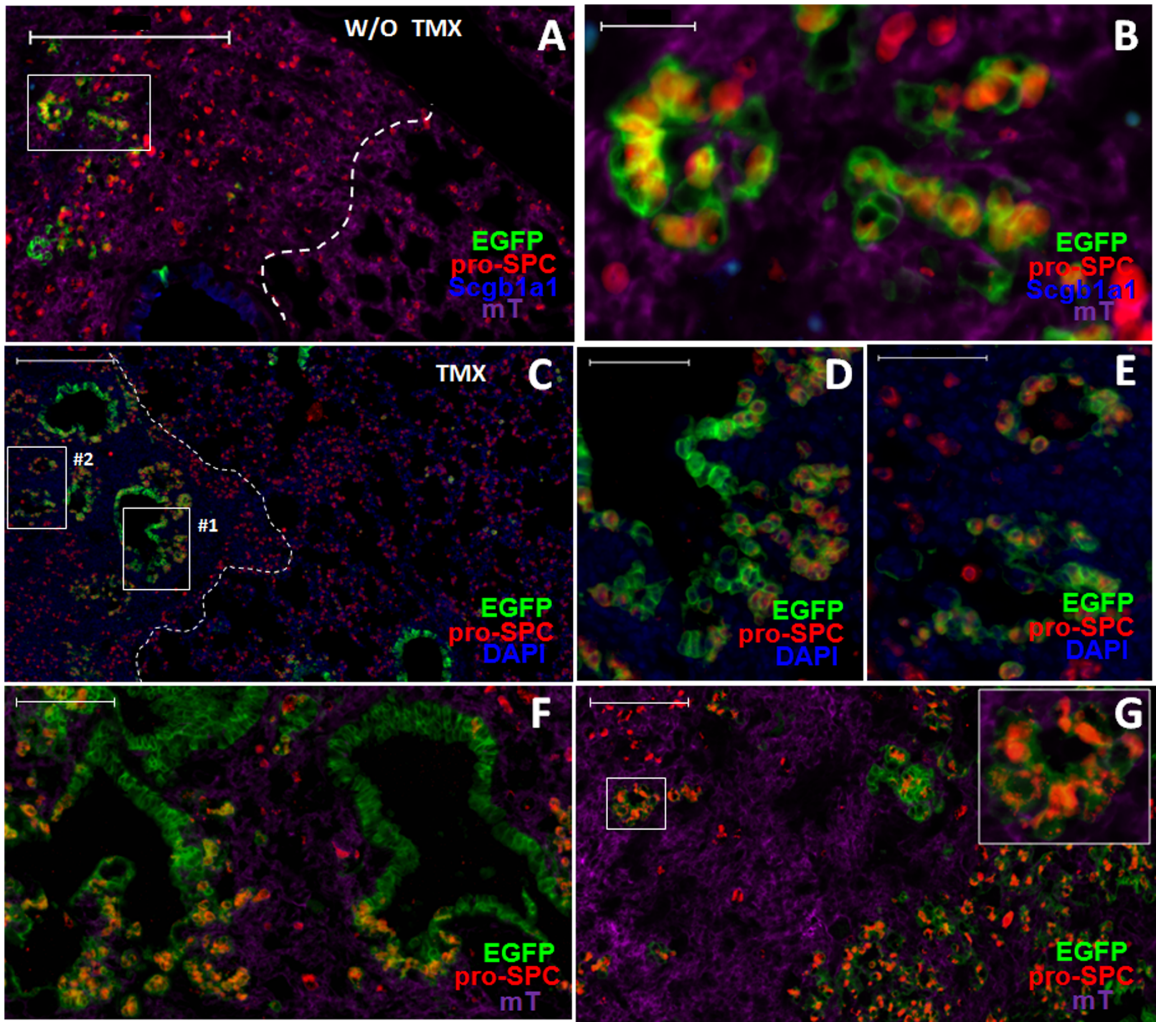
Clara cells give rise to pro-SPC^+^ cells in the ring structures. Scgb1a1-CreER:ACTB-mT-EGFP transgenic mice without TMX treatment (A&B) and with TMX treatment (C–G) were infected with influenza virus (A–E) or treated with bleomycin (F,G). (A–E) Shown are representative images of lung sections at 21 dpi analyzed for EGFP (green), pro-SPC (red), Scgb1a1 (blue), and tomato red (mT, purple). The white broken line in (A) and (C) demarcates the infiltrated area (to the left), and the normal area (to the right). Higher magnifications of the selected areas in (A) and (C) are shown as (B) and (D, E), respectively. The tomato red channel is shown in (A) and (B) but not in (C–E). (F,G) Representative images of lung sections at 21 day post bleomycin treatment analyzed for EGFP (green), pro-SPC (red) and tomato red (purple). Scale bars: (A,C) 200 µm; (F,G) 100 µm; (B,D,E) 20 µm.

To further test this possibility, we performed lineage tracing in Scgb1a1-CreER:ACTB-mT-EGFP transgenic mice. Without TMX treatment, most pro-SPC^+^ cells were EGFP^+^ in ∼16% of the ring structures in the damaged parenchyma 21 dpi (Figure 5A,B). Following TMX treatment, most pro-SPC^+^ cells were EGFP^+^ in ∼72% of the ring structures in the damaged parenchyma 21 dpi, suggesting that the pro-SPC^+^ cells in the ring structures are also originated from Clara cells. As in the wild-type mice, we frequently observed EGFP^+^ SBECs in the bronchioles that invaginated akin to the process of forming ring structures in transgenic mice following influenza infection (Figure 5C–E) and bleomycin treatment (Figure 5F,G). Together, these data suggest that the Clara cells give rise to pro-SPC^+^ cells in the ring structures through SBEC intermediates.

### Repair of Pulmonary Injury Shares Certain Features with Embryonic Lung Development

**Figure 6:**
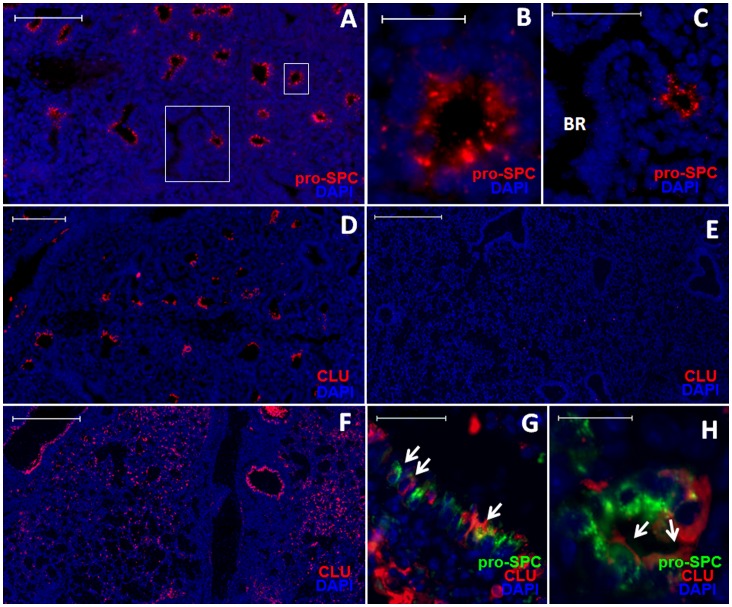
Repair of pulmonary damage exhibits features similar to embryonic lung development. A–C. Representative images of pro-SPC (red) and DAPI (blue) staining of a fetal lung at gestational day 15. Higher maganification images of the boxed areas in (A) are shown in (B) and (C). (B) The cuboidal shape of pro-SPC^+^ cells and (C) the tip of a developing bronchiole are evident. D. Representative image of immunofluorescent staining of clusterin (red) and DAPI (blue) in fetal lung sections at gestational day 16. E&F. Representative images of immunofluorescent staining of clusterin (red) and DAPI (blue) in the lung sections of adult mice (E) without infection or (F) at 9 dpi. G&H. Co-staining of clusterin (red), pro-SPC (green) and DAPI (blue) in the lung sections of mice (G) 9 or (H) 15 dpi. Arrows point to clusterin-positive SBECs in a bronchiole (G) or in a ring structure (H). Scale bars: (A, D) 100 µm; (B,H) 20 µm; (C,G) 50 µm; (E,F) 500 µm.

The observed pro-SPC^+^ ring structures in the damaged lung parenchyma are reminiscent of pro-SPC^+^ tubules in the embryonic lungs. At gestational day 15, the lung is in the pseudoglandular stage, and is filled with developing tubules. Many of the tubules are lined with pro-SPC^+^ cuboidal cells in ring structures (Figure 6A–C), similar to the pro-SPC^+^ ring structures observed in the damaged parenchyma (Figure 4B and Figure 5B). Clusterin is transiently expressed in the embryonic lung, where it promotes tissue morphogenesis [19], [20]. We also detected intense clusterin staining at the termini of the developing bronchioles during the pseudoglandular stage of embryonic lung development (Figure 6D). In contrast, virtually no cells were stained positive for clusterin in the adult lung (Figure 6E). However, following influenza infection, clusterin expression was induced in the lung (Figure 6F). Some SBECs, including those in the ring structures, stained positive for clusterin (Figure 6G,H). In contrast to the embryonic lung, the expression pattern of clusterin in the damaged lung was not restricted to the tips of bronchioles, but rather dispersed throughout the damaged area (Figure 6F). These findings suggest that the repair of damaged lung tissue in adult animals shares some features of the embryonic lung development, and that Scgb1a1^−^ SBECs in the infected adult lung appear to be phenotypically and functionally similar to pro-SPC^+^ cuboidal cells in the embryonic lung.

## Discussion

In the normal lung, Scgb1a1^+^ Clara cells reside in the bronchiolar epithelia, whereas pro-SPC^+^ AT2s reside in the alveolar epithelia. Only rarely are both Scgb1a1^+^ and pro-SPC^+^ cells (BASCs) observed at the BADJs [6], [21], [22]. However, following influenza virus infection or bleomycin treatment, pro-SPC^+^ cells (SBECs) were rapidly induced in the bronchioles in the damaged areas. Our kinetic study reveals that the SBECs initially expressed Scgb1a1 and exhibited morphology similar to Clara cells, but Scgb1a1 expression was lost as SBECs further differentiate. SBECs were unlikely to be derived from AT2s, since SBECs were only observed within damaged areas where AT2s were depleted (Figure 1C and Figure S1). Furthermore, no SBECs were induced following naphthalene treatment which does not cause significant damage to alveolar epithelia (i.e., despite the presence of AT2s). Although putative BASCs may contribute to Scgb1a1^+^ SBECs at BADJs, their rather low frequency (∼1%, Figure S2E) is unlikely to account for the rapid induction of SBECs throughout the bronchioles. Most importantly, our lineage tracing study provides definitive evidence that SBECs are induced from Clara cells following injury (Figure 3). Without TMX treatment, ∼10% of Clara cells were labeled with EGFP, while correspondingly ∼10% of SBECs were labeled with EGFP following alveolar damage. Upon TMX treatment, ∼80% of Clara cells were labeled with EGFP, while correspondingly ∼75% of SBECs were labeled. Clara cells are known to be heterogeneous and Clara^v^ cells play a critical role in repairing naphthalene-induced injury. Clara^v^ cells reside at BADJs or NEBs, and are negative for Cyp2f2 [4], [5], [18]. In contrast, most SBECs do not reside at BADJs and are positive for Cyp2f2, indicating that SBECs are not Clara^v^ cells per se. Although further studies are warranted to determine if SBECs are derived from Clara^V^ cells, our current findings strongly suggest that the majority of SBECs are likely derived from Clara cells.

Associated with the regeneration of alveolar epithelia, SBECs change their phenotype and anatomical locations. Initially, SBECs are localized in the bronchioles and express Scgb1a1. With time, SBECs lose Scgb1a1 expression and become pro-SPC^+^ cells in the damaged parenchyma. As with wild-type mice, SBECs also progress from Scgb1a1^+^ to Scgb1a1^−^ phenotype in the transgenic mice and these cells can be labeled sequentially with EGFP. It is possible that Clara cells might give rise to Scgb1a1^+^ and Scgb1a1^−^ SBECs separately. However, it is more likely that the Scgb1a1^−^ SBECs are derived from the Scgb1a1^+^ SBECs based on their sequential induction and that the decrease in the proportion of Scgb1a1^+^ SBECs over time is correlated with the increase in the proportion of Scgb1a1^−^ SBECs. We frequently observed ring structures containing pro-SPC^+^ cells in the damaged parenchyma. By lineage tracing, we show that the pro-SPC^+^ cells in the ring structures are derived from Clara cells. These pro-SPC^+^ ring structures in the damaged parenchyma resemble the pro-SPC^+^ tubules in the embryonic lungs. At gestational day 15 in the mouse, the lung is at the pseudoglandular stage and is filled with developing tubules. Many of these tubules contain pro-SPC^+^ multi-potent embryonic progenitor cells [23], [24], which eventually develop into both bronchiolar and alveolar epithelia [25], [26]. As in the embryonic lung, clusterin is re-expressed in the lung tissue including in SBECs following influenza infection. These observations underscore the similarity between alveolar epithelial development in the embryonic lung and alveolar epithelial regeneration following severe pulmonary damage. As we and others have shown previously, Scgb1a1^+^ cells, most likely Clara cells, give rise to AT2s to repair alveolar epithelia. The evidence presented here strongly suggest that this differentiation goes through SBECs, which invaginate to form pro-SPC^+^ ring structures and eventually regenerate alveolar epithelia in a process similar to embryonic alveolar development, even though we still lack the direct evidence like lineage tracing to show the differentiation from SBECs to AT2s. Differentiation of Clara cells to AT2s through the intermediate stage of SBECs during severe alveolar damage is compatible with the anatomical structure, because it would ensure that the newly regenerated alveolar spaces within damaged areas are connected to bronchioles.

Recently, different lung stem/progenitor cells, including p63^+^ cells in the distal airway [27], integrin α6β4^+^ cells [12] and BASCs [6], were reported to mediate the repair of severe pulmonary damages. In particular, the p63^+^ cells were shown to give rise to “pods” or ring-like structures in the damaged lung parenchyma following influenza infection [27]. By immunofluorescent staining, SBECs were negative for p63 (unpublished data), consistent with the observation that p63^+^ cells are distinct from pro-SPC^+^ cells in the damaged parenchyma [27]. While it is clear that SBECs are not p63^+^ cells, whether SBECs are derived from p63^+^ cells following influenza infection or bleomycin treatment remains to be determined. It is worth to note that even though p63^+^ cells can be labeled in Krt14-CreER transgenic mice, a lineage tracing has yet to be performed to verify their contribution to alveolar regeneration [27]. In another study, α6β4^+^ cells isolated from adult mouse lung were reported to give rise to Clara cells and AT2s in kidney capsules when mixed together with fetal lung cells [12]. Since ∼10% of the purified α6β4-positive cells used in the study were strongly positive for Scgb1a1 [12], these contaminating Clara cells could have given rise to Clara cells and AT2s in the engrafted renal capsule. Since the majority of α6β4^+^ cells were negative for Scgb1a1 or pro-SPC and resided at BADJs and alveoli, SBECs are probably not α6β4^+^ cells. Compared to BASCs, SBECs also express both Scgb1a1 and pro-SPC initially. However, the two cell types are quite different in a number of ways: BASCs reside at the BADJ, whereas the SBECs are found in both terminal and non-terminal bronchioles; BASCs are dimly immunopositive for pro-SPC, while SBECs are intensely positive for pro-SPC; although we detect BASCs at the BADJ in non-infected mice, their frequency is at least one order of magnitude lower than that of the SBECs. Finally, the Scgb1a1^−^ SBECs do not express CCSP, and thus cannot be the same cells as BASCs. Despite these differences, however, we cannot completely exclude the possibility that Scgb1a1^+^ SBECs at BADJs are the same cells as BASCs, and that the Scgb1a1^−^ SBECs are the progeny of BASCs en route to differentiate into AT2s.

Rawlins et al. [7] reported that following oxygen-induced alveolar damage, Clara cells and BASCs do not contribute significantly to alveolar epithelial repair, but they cautioned that their data do not exclude this possibility in case of severe alveolar damage. A comparison of lung damage and repair in different models is instructive. We show that while influenza infection and bleomycin treatment induces SBECs, naphthalene treatment, which does not induce alveolar damage, does not induce SBECs, suggesting that Clara cell to SBEC to AT2 differentiation is a common pathway induced by severe alveolar damage. It is possible that oxygen does not induce severe alveolar damage to induce SBECs.

Clara cells are known to give rise to new Clara cells as well as ciliated cells. We and others have recently reported that Clara cells also likely give rise to AT2s during the repair of severe alveolar damage. Evidence presented here strongly suggests that Clara cell to AT2 differentiation goes through an intermediate stage of SBECs.

## Materials and methods

### Animals, Influenza Infection and Chemical Treatments

C57BL/6 (B6) mice were purchased from the Center for Animal Resources, Singapore. Transgenic ACTB-mT-EGFP (stock number 007676) mice on the B6 background were purchased from the Jackson Laboratories. Scgb1a1-CreER transgenic mice were provided by Dr. Brigid Hogan of Duke University, USA. Scgb1a1-CreER:ACTB-mT-EGFP double transgenic mice were generated by breeding Scgb1a1-CreER mice with ACTB-mT-EGFP mice in the animal facility at the National University of Singapore (NUS). To induce Cre-mediated recombination, Scgb1a1-CreER:ACTB-mT-EGFP transgenic mice were treated with tamoxifen (TMX) in corn oil (or corn oil only as control) at the dose of 0.25 mg per gram body weight, every other day for four times [6]. TMX-treated mice were infected with influenza A/Puerto Rico/8/34 (PR8) virus or treated with bleomycin one week later (2 weeks after the initiation of TMX treatment). Mice at 8–12 weeks of age were infected with a sub-lethal dose of PR8 virus (100 pfu per mouse) or given bleomycin (0.5–1 U per kg body weight) by intratracheal instillation under anesthesia. All animals were housed in biosafety level 2 animal facilities at NUS.

### Ethics Statement

This study was carried out in strict accordance with the National Advisory Committee for Laboratory Animal Research (NACLAR) Guidelines (Guidelines on the Care and Use of Animals for Scientific Purposes) in facilities licensed by the Agri-Food and Veterinary Authority of Singapore (AVA), the regulatory body of the Singapore Animals and Birds Act. The protocol was approved by the Institutional Animal Care and Use Committee (IACUC), National University of Singapore. Mice were monitored every day after injury. At different time points, a group of mice were euthanatized by injection of ketamine/meditomidine, the lung samples were then collected for experiments. Any mouse with 30% body weight loss will be euthanatized immediately.

All other experimental protocols are described in the Supplementary Methods in File S1.

## Supporting Information

Figure S1 **A model of influenza virus-induced lung damage and repair.** A. Relative body weight (means ± S.E.) of mice (n = 5 per group) at the indicated dpi. B. Viral titers in the lung at different dpi. The levels of influenza nucleoprotein (NP) RNA in the lung tissues were quantified by real-time RT-PCR. Data were normalized to the levels of ribosomal protein L32 RNA, and were then expressed as fold changes (means ± S.E.) over that for 1 dpi (n = 3 mice per time-point). C. Representative H&E stains of lung sections of uninfected control and infected mice at the indicated dpi. D&E. Representative immunofluorescent images of lung sections stained for influenza virus (red), Scgb1a1 (green) and DAPI (blue); or for influenza virus (red), pro-SPC (green) and DAPI (blue) at 3 dpi. Arrows point to cells positive for (D) both Scgb1a1 and influenza virus, or (E) for both pro-SPC and influenza virus. Arrowheads point to (D) Clara cells that sloughed off from the bronchiolar epithelium, or (E) the influenza virus single-positive cells in alveolar epithelia. F. Representative immunofluorescent images of lung sections stained for Scgb1a1 (green), pro-SPC (red), and DAPI (blue) at 7 dpi. AL, alveolar; BR, bronchiole. The broken white line demarcates the infiltrated area (upper) from the normal area (lower) of the lung. G&H. Transgenic rCCSP-rtTA:tetO-Cre:ACTB-mT-EGFP mice were given water containing doxycycline for 7 days, and then infected intra-tracheally with influenza virus. At 7 dpi, lung sections were analyzed for EGFP (green), pro-SPC (red), and DAPI (blue). Shown are representative images of lung sections of (G) uninfected and h) infected mice. For better visualization of immunofluorescence, the tomato red channel is not shown. I. Representative images of co-staining for Ki67 (green), Scgb1a1 (red) and DAPI (blue) in lung sections of mice at 9 dpi. Arrows indicate cells that are double-positive for Scgb1a1 and Ki67. Scale bars: (C) 1000 µm; (D,E) 50 µm; (F) 500 µm; (G,H) 100 µm.
(TIF)

Figure S2 **SBECs are not induced following naphthalene treatment.** A&B. Representative Scgb1a1 (green), pro-SPC (red) and DAPI (blue) staining of lung sections of mice at 6 days after naphthalene treatment. Arrows in (B) indicate putative BASCs at BADJs. C&D. Representative staining for Scgb1a1 (red), Ki67 (green) and DAPI (blue) of lung sections from mice at 9 (C) or 12(D) days after naphthalene treatment. Arrows indicate the Scgb1a1 and Ki67 double-positive cells. E. Percentages (means ± S.E.) of BADJs containing BASCs or SBECs in mice without treatment (control) or at different days post naphthalene or bleomycin treatment. The numbers indicate the numbers of BADJ and mice from which the data were obtained. Scale bars: (A) 100 µm; (B–D) 50 µm.
(TIF)

Figure S3 **EGFP-positive Clara cells tend to cluster together in infected mice without TMX treatment.** Scgb1a1-CreER:ACTB-mT-EGFP transgenic mice without TMX treatment were infected with influenza virus. Shown are representative images of lung sections from mice at 21 dpi analyzed for expression of EGFP (green), and stained for Scgb1a1 (red) and DAPI (blue). The tomato red channel is not shown. Higher magnification image of the selected area in (A) is shown as (B). Scale bars: (A) 500 µm; (B) 50 µm.
(TIF)

Figure S4 **SBECs are positive for Cyp2f2.** A&B. Representative image of Cyp2f2 (green), pro-SPC (red) and DAPI (blue) staining of lung sections of mice at 9 days post influenza virus infection. Scale bars: (A,B) 20 µm.
(TIF)

Figure S5 **Pro-SPC^+^ cells in damaged parenchyma or ring structures are rarely in proliferation.** A–C. Representative images of Scgb1a1 (red, A ) or pro-SPC (red, B&C), BrdU (Green) and DAPI (blue) staining of lung tissue sections of mice at 9 (A) or 17 (B&C) dpi. Arrows indicate BrdU positive Clara cells (A) or Pro-SPC^+^ cells in damaged parenchyma (B). D. Percentages of BrdU^+^ pro-SPC^+^ cells in the ring structures. Mice infected with influenza virus were sacrificed at different days post infection. The numbers indicate the number of cells and mice from which the data were obtained. Scale bars: (A&C) 50 µm; (B) 100 µm.
(TIF)

File S1 (DOCX)
