# Supplementary figures and images for: A Cellular Pathway Involved in Clara Cell to Alveolar Type II Cell Differentiation after Severe Lung Injury
Source: PLoS One. 2013 Aug 5;8(8):e71028. doi: 10.1371/journal.pone.0071028 (PMC3734298; doi:10.1371/journal.pone.0071028)

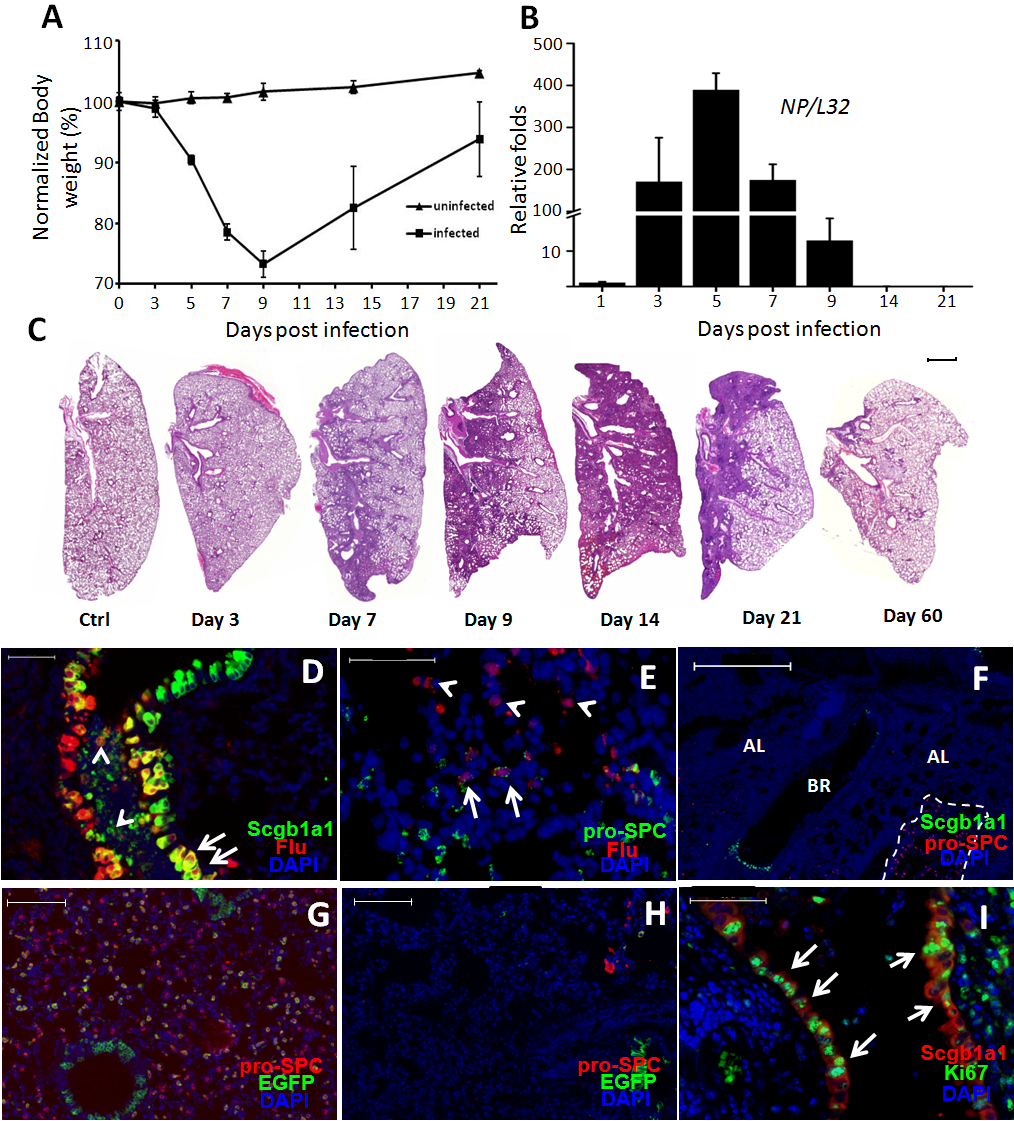

Supplement: Figure S1 — A model of influenza virus-induced lung damage and repair. A. Relative body weight (means ± S.E.) of mice (n = 5 per group) at the indicated dpi. B. Viral titers in the lung at different dpi. The levels of influenza nucleoprotein (NP) RNA in the lung tissues were quantified by real-time RT-PCR. Data were normalized to the levels of ribosomal protein L32 RNA, and were then expressed as fold changes (means ± S.E.) over that for 1 dpi (n = 3 mice per time-point). C. Representative H&E stains of lung sections of uninfected control and infected mice at the indicated dpi. D&E. Representative immunofluorescent images of lung sections stained for influenza virus (red), Scgb1a1 (green) and DAPI (blue); or for influenza virus (red), pro-SPC (green) and DAPI (blue) at 3 dpi. Arrows point to cells positive for (D) both Scgb1a1 and influenza virus, or (E) for both pro-SPC and influenza virus. Arrowheads point to (D) Clara cells that sloughed off from the bronchiolar epithelium, or (E) the influenza virus single-positive cells in alveolar epithelia. F. Representative immunofluorescent images of lung sections stained for Scgb1a1 (green), pro-SPC (red), and DAPI (blue) at 7 dpi. AL, alveolar; BR, bronchiole. The broken white line demarcates the infiltrated area (upper) from the normal area (lower) of the lung. G&H. Transgenic rCCSP-rtTA:tetO-Cre:ACTB-mT-EGFP mice were given water containing doxycycline for 7 days, and then infected intra-tracheally with influenza virus. At 7 dpi, lung sections were analyzed for EGFP (green), pro-SPC (red), and DAPI (blue). Shown are representative images of lung sections of (G) uninfected and h) infected mice. For better visualization of immunofluorescence, the tomato red channel is not shown. I. Representative images of co-staining for Ki67 (green), Scgb1a1 (red) and DAPI (blue) in lung sections of mice at 9 dpi. Arrows indicate cells that are double-positive for Scgb1a1 and Ki67. Scale bars: (C) 1000 µm; (D,E) 50 µm; (F) 500 µm; (G,H) 100 µm. (TI [file pone.0071028.s001.tif]

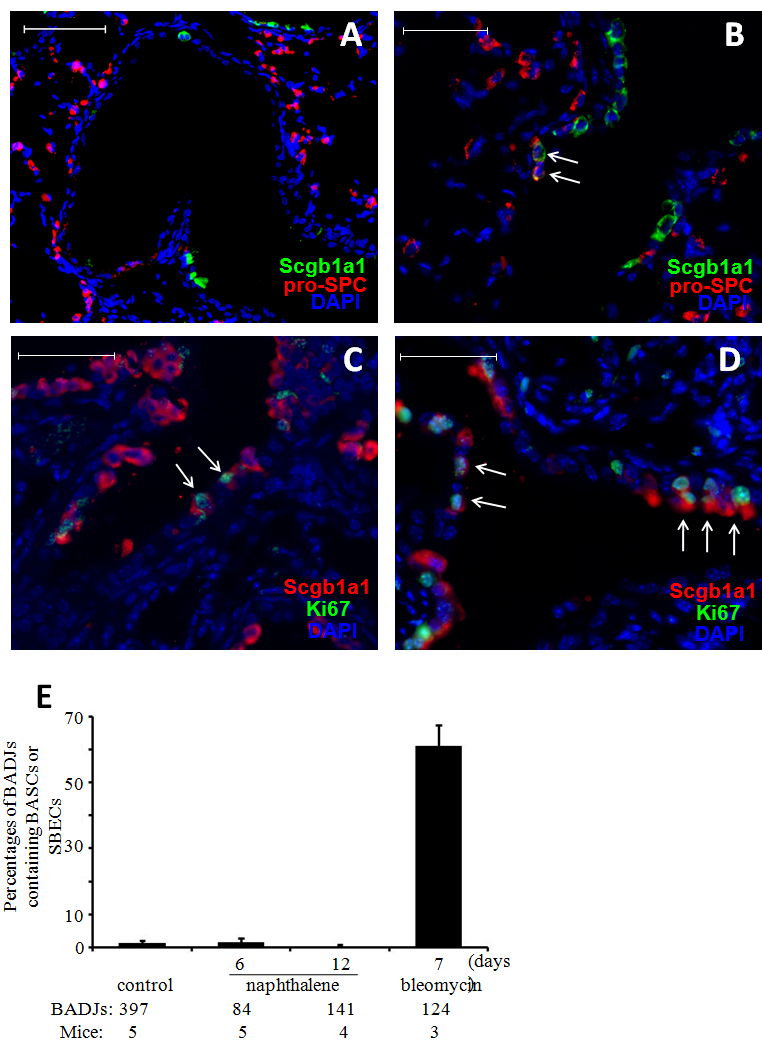

Supplement: Figure S2 — SBECs are not induced following naphthalene treatment. A&B. Representative Scgb1a1 (green), pro-SPC (red) and DAPI (blue) staining of lung sections of mice at 6 days after naphthalene treatment. Arrows in (B) indicate putative BASCs at BADJs. C&D. Representative staining for Scgb1a1 (red), Ki67 (green) and DAPI (blue) of lung sections from mice at 9 (C) or 12(D) days after naphthalene treatment. Arrows indicate the Scgb1a1 and Ki67 double-positive cells. E. Percentages (means ± S.E.) of BADJs containing BASCs or SBECs in mice without treatment (control) or at different days post naphthalene or bleomycin treatment. The numbers indicate the numbers of BADJ and mice from which the data were obtained. Scale bars: (A) 100 µm; (B–D) 50 µm. (TIF) [file pone.0071028.s002.tif]

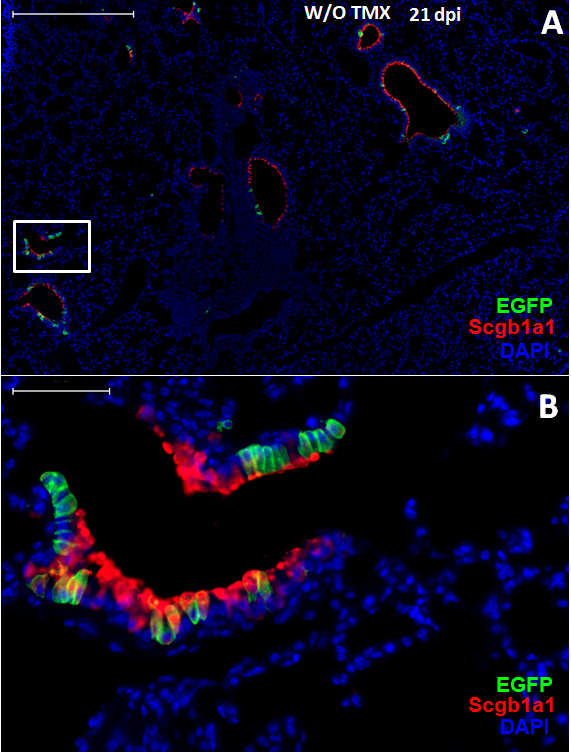

Supplement: Figure S3 — EGFP-positive Clara cells tend to cluster together in infected mice without TMX treatment. Scgb1a1-CreER:ACTB-mT-EGFP transgenic mice without TMX treatment were infected with influenza virus. Shown are representative images of lung sections from mice at 21 dpi analyzed for expression of EGFP (green), and stained for Scgb1a1 (red) and DAPI (blue). The tomato red channel is not shown. Higher magnification image of the selected area in (A) is shown as (B). Scale bars: (A) 500 µm; (B) 50 µm. (TIF) [file pone.0071028.s003.tif]

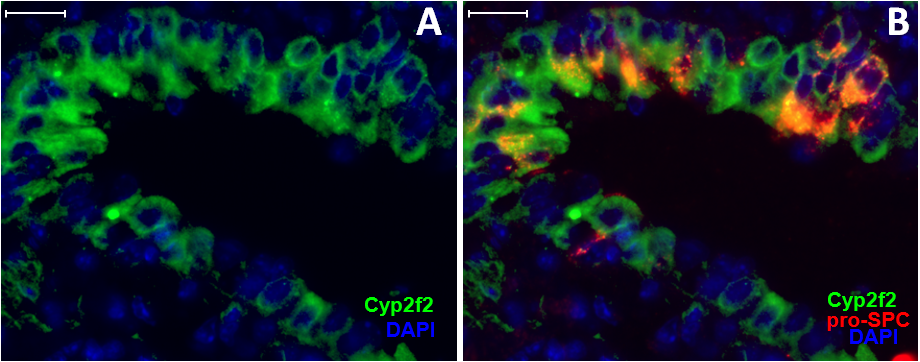

Supplement: Figure S4 — SBECs are positive for Cyp2f2. A&B. Representative image of Cyp2f2 (green), pro-SPC (red) and DAPI (blue) staining of lung sections of mice at 9 days post influenza virus infection. Scale bars: (A,B) 20 µm. (TIF) [file pone.0071028.s004.tif]

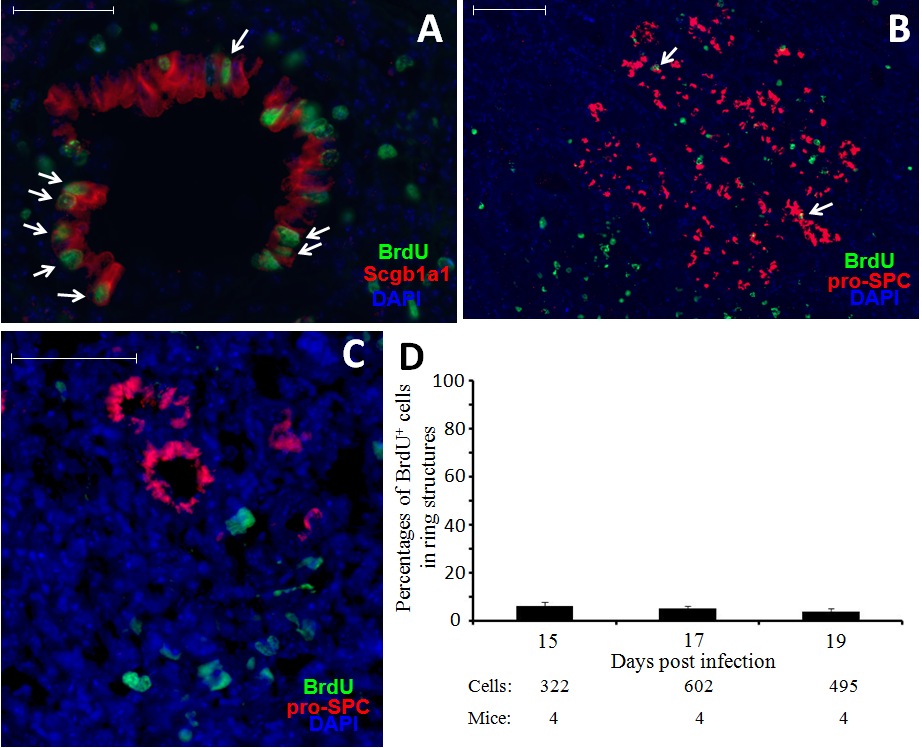

Supplement: Figure S5 — Pro-SPC+ cells in damaged parenchyma or ring structures are rarely in proliferation. A–C. Representative images of Scgb1a1 (red, A ) or pro-SPC (red, B&C), BrdU (Green) and DAPI (blue) staining of lung tissue sections of mice at 9 (A) or 17 (B&C) dpi. Arrows indicate BrdU positive Clara cells (A) or Pro-SPC+ cells in damaged parenchyma (B). D. Percentages of BrdU+ pro-SPC+ cells in the ring structures. Mice infected with influenza virus were sacrificed at different days post infection. The numbers indicate the number of cells and mice from which the data were obtained. Scale bars: (A&C) 50 µm; (B) 100 µm. (TIF) [file pone.0071028.s005.tif]
