# Supplementary material for: A Cellular Pathway Involved in Clara Cell to Alveolar Type II Cell Differentiation after Severe Lung Injury
Source: PLoS One. 2013 Aug 5;8(8):e71028. doi: 10.1371/journal.pone.0071028 (PMC3734298; doi:10.1371/journal.pone.0071028)
Supplement: File S1 — (DOCX) [file pone.0071028.s006.docx]

**Supplementary results**

**An influenza-induced pulmonary damage and repair model**

To study how lung tissue is regenerated following severe damage, we developed a model of influenza virus-induced lung injury and repair, in which C57BL/6 (B6) mice were infected intra-tracheally with a sublethal dose of influenza A/Puerto Rico/8/34 H1N1 virus (PR8). Infected mice started to lose weight at 3 days post-infection (dpi), reached peak weight loss of ~25% by 9 dpi, and then gradually recovered (Figure S1A). The viral load in the lung reached maximal levels at 5 dpi, decreasing thereafter to undetectable levels at 14 dpi (Figure S1B). Hematoxylin and eosin (H&E) staining of lung tissue sections revealed post-infection infiltration of inflammatory cells, which peaked around 14 dpi, decreased significantly by 21 dpi, and was almost completely resolved by 60 dpi (Figure S1C).

As early as 3 dpi, both Clara cells and AT2s were stained positive for influenza virus (Figure S1D,E, arrows). Sloughing off of epithelial cells from bronchioles was evident in the infected areas of the lung (Figure S1D, arrowheads). By 7 dpi, the damage was so severe that most bronchioles in the infected areas (indicated by dense DAPI staining of nuclei of infiltrating cells) were depleted of Scgb1a1^+^ Clara cells, while the infiltrated parenchyma was almost completely devoid of pro-SPC^+^ AT2s (Figure S1F).

To determine whether the AT2s were depleted or whether they switched off expression of pro-SPC after infection, we utilized rCCSP-rtTA:tetO-Cre:ACTB-mT-EGFP triple transgenic mice. In this system, transcription of the rtTA transactivator is under the control of the rat Scgb1a1 promoter, transcription of Cre is under the control of tetO and doxycycline, while transcription of a membrane-bound form of tomato red (mT) or EGFP is under the control of the beta-actin promoter. All cells express tomato red without Cre-mediated recombination [[1](#_ENREF_1)], but only Scgb1a1^+^ cells, which are mostly Clara cells, switch to EGFP expression after doxycycline treatment and recombination. EGFP and mT signals could be directly detected without any staining. Because of leakage of the system, many AT2s were also induced to express EGFP after doxycycline treatment (Figure S1G). However, as early as 7 dpi, no pro-SPC^+^ or EGFP^+^ cells were detected in the infiltrated parenchyma (Figure S1H). Since EGFP is driven by the beta-actin promoter, these results suggest that AT2s are also infected by influenza virus, and are depleted from infected areas of the lung. Thus, influenza infection induces severe damage in both bronchiolar and alveolar epithelia.

Proliferation and differentiation of progenitor cells are necessary for tissue repair. We used Ki67 staining to evaluate proliferation of Scgb1a1^+^ and pro-SPC^+^ cells at various time-points following influenza infection. As early as 5 dpi, some Scgb1a1^+^ cells in the bronchiolar epithelia were Ki67^+^ (data not shown). By 9 dpi, almost all Scgb1a1^+^ cells in the damaged area were positive for Ki67 in some bronchioles (Figure S1I), indicating extensive proliferation. By 14 dpi, most bronchioles were covered with Scgb1a1^+^ Clara cells (data not shown). In contrast, very few pro-SPC^+^ AT2s were Ki67^+^ throughout the course of infection (data not shown). By 21-60 dpi, as infiltrated inflammatory cells in the damaged areas of the lung gradually disappeared, apparently normal looking alveolar structures appeared (Figure S1C). Collectively, these results indicate that influenza infection causes severe lung tissue damage in mice, which is repaired as the animals recover from the infection.

**Supplementary materials and methods**

**Mice and chemical treatment.** Transgenic rCCSP-rtTA (stock number 006232), tetO-Cre (stock number 006234) and ACTB-mT-EGFP (stock number 007676) mice on the B6 background were purchased from the Jackson Laboratories. The transgenic mice were bred to generate triple transgenic mice rCCSP-rtTA:tetO-Cre:ACTB-mT-EGFP in the animal facility at the National University of Singapore (NUS). The genotype of transgenic mice was determined according to the protocol from the Jackson Laboratories. To induce the recombination, triple transgenic mice were feed with water containing doxycycline (1mg/ml) for 7 days before experiment. For treating mice with naphthalene, naphthalene dissolved in corn oil was injected intraperitoneally into mice with a dose of 200mg per kg body weight. BrdU solution (BD Pharmingen) was injected intraperitoneally 2 hours before sacrifice at a dose of 50mg/kg body weight. All animals were housed in biosafety level 2 (BSL2) animal facilities at NUS. All animal protocols were approved by the Institutional Animal Care and Use Committees at US and Massachusetts Institute of Technology.

**Antibodies.** Polyclonal rabbit anti-Scgb1a1 antibody (US Biological, C5828) was used at 1:200 dilution. Polyclonal goat anti-Scgb1a1 (Santa Cruz Biotechnology, sc-9772), goat anti-pro-SPC (Santa Cruz Biotechnology, sc-7706), rabbit anti-pro-SPC (Santa Cruz Biotechnology, sc-7705), goat anti-clusterin (R&D Systems, AF2747), monoclonal rat anti-Ki67 (Dako, M7249) and mouse anti-BrdU (Leica Microsystems, NCL-BrdU) were used at 1:50 dilution. Rabbit anti-influenza virus serum was kindly provided by Dr. T. Narasaraju (NUS). Secondary antibodies (including donkey anti-rabbit, anti-goat, anti-rat, or anti-mouse) each with different Alexa Fluor conjugations were all purchased from Life Technologies, and used at 1:200 dilution.

**Histopathology and immunochemical staining.** Mice were euthanized at indicated post-infection time-points, lung tissues were harvested and fixed in 10% neutral buffered formalin solution (Sigma-Aldrich) for 24 hours. The tissues were processed with Tissue Processor (Leica Microsystems) and embedded in paraffin. Sections were cut at 5 μm thickness, mounted on polylysine-coated slides (Thermo Fisher Scientific), de-waxed, rehydrated, and processed for hematoxylin and eosin (H&E) staining according to a standard protocol. For immunofluorescent staining, antigen retrieval was carried out by either microwaving the slides in 0.01 M sodium citric acid buffer (pH 6.0) for 30 min (for anti-Ki67), or by digestion in 20 mg/ml proteinase K solution (in 50mM Tris-Cl, 1 mM EDTA, pH 8.0) at 37oC for 30 min (for all other antibodies). For BrdU staining, sections were processed according to the manufacturer’s protocol. Sections were then immersed for 1 hour in blocking buffer (3% BSA, 0.2% Triton X-100 in PBS), then incubated in primary antibody (made up in blocking buffer) at 4^o^C overnight, followed by incubation with secondary antibody at 4^o^C for 1 hour. All lung sections were counterstained with DAPI, mounted with antifade reagent (Life Technologies), and then scanned with a high-resolution MIRAX MIDI system (Carl Zeiss) equipped with both bright field and fluorescence illumination. Images were analyzed by the MIRAX Viewer software.

**RNA extraction and real-time fluorescence quantitative PCR.** Mice were euthanized at indicated time-points post-infection, and total RNAs were extracted from lung tissues with TRIZOL reagent (Life Technologies) according to the manufacturer’s protocol. Two micrograms of total RNA from each sample was reverse-transcribed using oligo(dT) primers and Superscript II reverse transcriptase (Life Technologies). PCR was performed with SsoFast EvaGreen Supermix using the CFX96 real-time PCR detection system (Bio-Rad Laboratories). Primer sequences for the L32 housekeeping gene and influenza virus N gene were as previously described [[2](#_ENREF_2),[3](#_ENREF_3)].

**Supplementary references**

1. Muzumdar MD, Tasic B, Miyamichi K, Li L, Luo L (2007) A global double-fluorescent Cre reporter mouse. Genesis 45: 593-605.

2. Doyle SE, O'Connell R, Vaidya SA, Chow EK, Yee K, et al. (2003) Toll-like receptor 3 mediates a more potent antiviral response than Toll-like receptor 4. J Immunol 170: 3565-3571.

3. Alt JA, Bohnet S, Taishi P, Duricka D, Obal F, Jr., et al. (2007) Influenza virus-induced glucocorticoid and hypothalamic and lung cytokine mRNA responses in dwarf lit/lit mice. Brain Behav Immun 21: 60-67.
